# The serotonin receptor 7 and the structural plasticity of brain circuits

**DOI:** 10.3389/fnbeh.2014.00318

**Published:** 2014-09-12

**Authors:** Floriana Volpicelli, Luisa Speranza, Umberto di Porzio, Marianna Crispino, Carla Perrone-Capano

**Affiliations:** ^1^Department of Pharmacy, University of Naples Federico IINaples, Italy; ^2^Institute of Genetics and Biophysics “Adriano Buzzati Traverso”, National Research Council (CNR)Naples, Italy; ^3^Department of Biology, University of Naples Federico IINaples, Italy

**Keywords:** 5-HT7R, brain connectivity, brain development, neurodevelopmental diseases, neuronal cytoarchitecture, serotonin

## Abstract

Serotonin (5-hydroxytryptamine, 5-HT) modulates numerous physiological processes in the nervous system. Together with its function as neurotransmitter, 5-HT regulates neurite outgrowth, dendritic spine shape and density, growth cone motility and synapse formation during development. In the mammalian brain 5-HT innervation is virtually ubiquitous and the diversity and specificity of its signaling and function arise from at least 20 different receptors, grouped in 7 classes. Here we will focus on the role 5-HT7 receptor (5-HT7R) in the correct establishment of neuronal cytoarchitecture during development, as also suggested by its involvement in several neurodevelopmental disorders. The emerging picture shows that this receptor is a key player contributing not only to shape brain networks during development but also to remodel neuronal wiring in the mature brain, thus controlling cognitive and emotional responses. The activation of 5-HT7R might be one of the mechanisms underlying the ability of the CNS to respond to different stimuli by modulation of its circuit configuration.

## Serotonin and brain development

Serotonin is a neurotransmitter modulating numerous physiological processes in the nervous system such as sleep, mood, aggressive behavior, sexual behavior, sensory processing, cognitive control, emotion regulation, autonomic responses, and motor activity (for reviews see Daubert and Condron, 2010; Lesch and Waider, 2012). 5-HT was first discovered in the gut and enterochromaffin cells by the pharmacologist V. Erspamer in the middle thirties and subsequently in blood serum as a vasoconstrictor, hence the name serotonin (serum that gives tone; Rapport et al., 1948).

Serotonergic neurons are found in a variety of organisms, from C. Elegans to vertebrates. In mammals, they are among the earliest neurons being differentiated during development, and comprise a widely distributed neuronal network in the brain (Lesch and Waider, 2012).

Several experimental data have indicated that 5-HT may act as a signaling cue in the fetal brain during critical periods of development. It is recognized that 5-HT is synthesized early in embryonic development and its receptors are early expressed. In addition to the endogenous 5-HT, the brain of the fetus receives it also from the placenta of the mother, further emphasizing the importance of 5-HT in the early embryonic development of the brain. The contribution of these maternal-placental-fetal interactions appears to be critical for brain circuit wiring and for long-term brain functions (Bonnin et al., 2011). In particular, the 5-HT system plays a crucial role in the establishment of cortical circuits by controlling key cellular processes including neuronal migration and dendritic differentiation (Puig and Gulledge, 2011; Vitalis et al., 2013; Dayer, 2014). Cortical circuits control cognitive processes and their function is highly dependent on their structure that is shaped during development. Along this line, alteration of the 5-HT signaling system is associated to neurodevelopmental disorders affecting cognitive abilities, as mentioned below. Studies using genetic mouse models reveal that excessive 5-HT levels in the brain alter the correct development of mouse somatosensory cortex (Cases et al., 1996; Persico et al., 2001; Dayer, 2014). On the other hand, the depletion of 5-HT in the brain leads to behavioral and functional deficits, despite the lack of detectable cellular or morphological alterations in the CNS (Hendricks et al., 2003; Savelieva et al., 2008; Alenina et al., 2009). The absence of CNS evident morphological defects in these mouse models suggests that the lack of brain 5-HT may only affect fine shaping of specific circuits, so that these alterations are not revealed by gross morphological analyses of brain. On the other hand, transgenic mice with a 75% reduction in brain 5-HT levels showed reduced brain growth and delayed cortical maturation during postnatal life (Narboux-Nême et al., 2013). Interestingly, in a recent elegant experiment it was demonstrated that lack of brain 5-HT produced a striking reduction of serotonergic innervation in diencephalic areas (the suprachiasmatic and thalamic paraventricular nuclei) and a marked serotonergic hyperinnervation in forebrain areas (nucleus accumbens and hippocampus; Migliarini et al., 2013). These data strongly suggest that 5-HT can either promote or inhibit terminal arborization of serotonergic axons depending on specific targets in the brain. In addition, these findings confirm that alterations of 5-HT levels during CNS development produce severe abnormalities in the serotonergic circuitry affecting the proper wiring of the brain. Consistently, numerous studies from both vertebrate and invertebrate organisms support the idea that 5-HT regulates neurite outgrowth and establishment of neuronal connectivity during brain development (Daubert and Condron, 2010; Lesch and Waider, 2012), and that alterations in early serotonin signaling may produce long-lasting changes. The latter might be the basis of a number of neuropsychiatric disorders which likely have developmental origins, such as schizophrenia, depression, affective disorders, anxiety and autism (Lesch and Waider, 2012; Velasquez et al., 2013; Dayer, 2014). Nevertheless, the key molecular and cellular events through which the 5-HT signaling affects brain connectivity are still poorly investigated. The high number of the 5-HT receptors and the lack of selective pharmacological agonists or inhibitors for the various subtypes have hampered a detailed analysis on their selective involvement in shaping brain networks and modulating neuronal cytoarchitecture. Actually, as mentioned, in the mammalian brain serotonergic neurons exert their effects through 20 subtypes of receptors that are grouped in 7 distinct classes based on pharmacological properties, amino acid sequences, gene organization, and their coupled second messenger pathways. All 5-HT receptors, with the exception of the 5-HT3, are typical G-protein-coupled-receptors (GPCRs) with seven transmembrane domains. The 5-HT3 receptor, instead, is a ligand-gated ion channel. This great diversity of receptors indicates the wide physiological role of 5-HT in the nervous system, whose complexity still needs to be elucidated (Pytliak et al., 2011; Gellynck et al., 2013).

## The serotonin receptor 7

The 5-HT7 receptor was cloned independently by three laboratories in 1993 (Bard et al., 1993; Lovenberg et al., 1993; Ruat et al., 1993). A number of functional splice variants of this receptor have been identified due to the presence of introns in the 5-HT7R gene (Gellynck et al., 2013).

**Figure 1:**
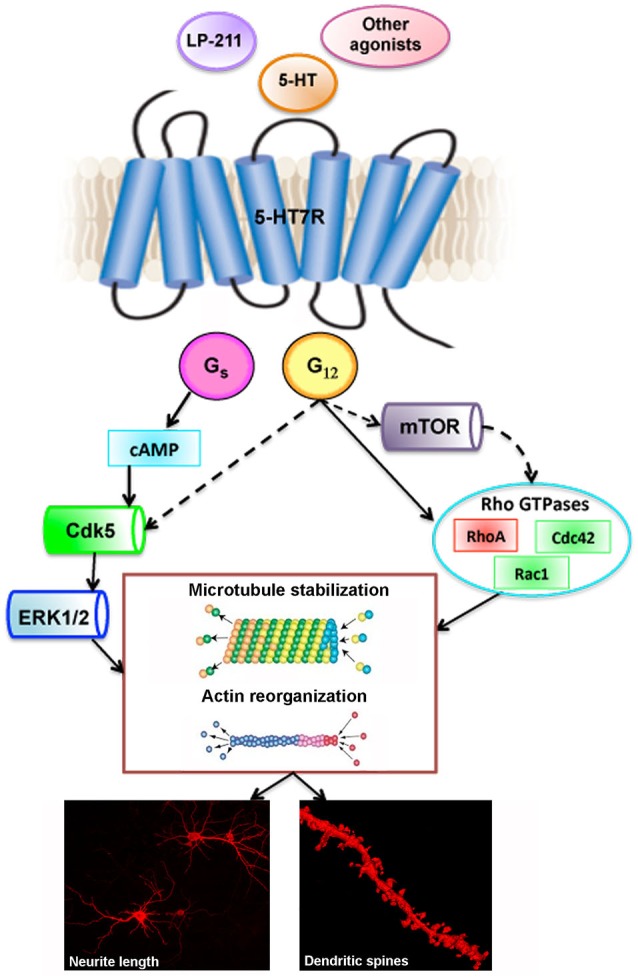
**Stimulation of 5-HT7R: schematic drawing of signaling pathways and downstream effectors leading to remodeling of neuronal morphology**. Full lines indicate established pathways, dashed lines indicate putative targets. Lower panels are photomicrographs from DiI-labeled striatal neurons in culture (left panel), and striatal slices from brain adult mice (right panel, 3D- reconstruction). 5-HT: 5-hydroxytryptamine; 5-HT7R: serotonin receptor 7; LP-211: selective 5-HT7R agonist; Cdk5: cyclin-dependent kinase 5; ERK 1/2: extracellular signal-regulated kinases 1/2; mTOR: mammalian target of rapamycin.

The 5-HT7R activates Gα_s_ that stimulates adenylate cyclase, resulting in an increase in cAMP. The cAMP activates protein kinase A (PKA) leading to phosphorylation of different proteins (Leopoldo et al., 2011). The 5-HT7R is also coupled to stimulation of the mitogen-activated protein kinase extracellular signal-regulated kinases (ERK; Errico et al., 2001). More recently, the 5-HT7R has also been shown to interact with another member of the G protein family, the Gα_12_. Activation of the 5-HT7R/Gα_12_ signaling pathway leads to stimulation of Rho GTPases, Cdc42 and RhoA (Kvachnina et al., 2005; Figure 1).

The 5-HT7R is expressed in both the CNS and in peripheral tissues. In the CNS the receptor is expressed in the diencephalon, forebrain and in the Purkinje neurons of the cerebellum (for reviews see Matthys et al., 2011; Gellynck et al., 2013). The wide distribution of 5-HT7R in the brain reflects the numerous functions in which this receptor is implicated, such as circadian rhythms, sleep-wake cycle, thermoregulation (Leopoldo et al., 2011; Adriani et al., 2012; Monti et al., 2014; Romano et al., 2014) and nociception (Garcia et al., 2011), but also cognitive functions such as learning and memory processing (Roberts and Hedlund, 2012; Freret et al., 2014; Meneses, 2014). Importantly, the putative involvement of 5-HT7R in many neuropathological processes such as anxiety, schizophrenia, epilepsy, migraine, impulsivity and depression, cognitive and mood disturbances (Hedlund, 2009; Cates et al., 2013; Gellynck et al., 2013), makes it a potential target for new therapeutic applications.

To explain the fact that a single receptor is involved in such a variety of physio-pathological processes of the CNS, the GPCR dimerization has been proposed as a possible key mechanism that introduces diversity in 5-HT7 receptor signaling (Matthys et al., 2011). Accordingly, in a recent study, it has been shown that heterodimerization of 5-HT7 and 5-HT1A receptors differentially regulates these receptors signaling and trafficking (Renner et al., 2012).

## The serotonin receptor 7 shapes neuronal circuits during development

Numerous recent data indicate that the activation of 5-HT7R modulates neuronal morphology, excitability and plasticity contributing to the establishment of brain connectivity during embryonic and early postnatal life.

Using fibroblast cell lines, it was demonstrated that serotonin stimulation of 5-HT7R induces filopodia formation and cell rounding through interaction of the receptor with Gα_12_ subunit of heterotrimeric G-protein and activation of Rho GTPases. This finding prompted more detailed studies using mouse hippocampal neurons in culture, where activation of the endogenous 5-HT7R, using the agonist 5-CT, determines pronounced extension of neurite length (Kvachnina et al., 2005). These results have been confirmed by more recent analyses where 5-HT7R was stimulated with 5-HT, or with the selective agonist AS19, or with the 5-HT_1A/7_ receptor agonist 8-OH-DPAT (Tajiri et al., 2012; Rojas et al., 2014). The effects of the 5-CT, as well as those of the 8-OH-DPAT were abolished by the co-treatment with 5-HT7R selective antagonist SB-269970, indicating the specific involvement of 5-HT7R. The morphogenic effects of the 5-HT7R on neuronal cytoarchitecture have been demonstrated also for neurons from additional CNS areas. Treatment of cultured embryonic neurons from rodent striatal complex and cortex with 8-OH-DPAT and with the highly potent and selective 5-HT7R agonist LP-211, significantly enhances neurite outgrowth through pathways involving Cdk5 and extracellular signal-regulated kinases 1/2 (ERK). These effects are selectively due to the 5-HT7R stimulation since they are blocked by SB-269970. Neurite elongation requires de novo protein synthesis and is accompanied by qualitative and quantitative modifications of selected cytoskeletal proteins (Speranza et al., 2013). These findings delineate an overall picture of potential intracellular pathways and molecular mechanisms that underlie modulation of neuronal morphology due to 5-HT7R stimulation (Figure 1).

In accordance with the morphogenic role of 5-HT7R, it was demonstrated that prolonged stimulation of the 5-HT7R/Gα_12_ signaling pathway in early postnatal cultured hippocampal neurons leads to an increased number of dendritic protrusions and synaptic contacts, and enhances spontaneous synaptic activity. A similar morphogenic function of the 5-HT7R was confirmed in organized brain circuitries (organotypic slices preparation from the hippocampus of juvenile mice), where stimulation of 5-HT7R/Gα_12_ signaling pathway potentiates formation of dendritic spines, increases neuronal excitability and modulates synaptic plasticity (LTP). The latter effect was age-dependent, indeed it was observed in 1 week-old mice but not in adult animals, probably due to decreased hippocampal expression of the 5-HT7R during later post-natal stages (Kobe et al., 2012).

Intriguingly, 5-HT7R modulates NMDA receptors activity in hippocampal neurons. Long term activation of the 5-HT7R by the selective agonist LP 12 inhibits glutamate receptor signaling preventing NMDA-induced neurotoxicity (Vasefi et al., 2013a), while acute activation of 5-HT7R promotes NMDA receptor activity (Vasefi et al., 2013b).

In addition, activation of 5-HT7R modulates long-term depression mediated by metabotropic glutamate receptors in wild-type as well as in a mouse model of Fragile X- syndrome (FXS; Costa et al., 2012). These animals exhibit spatial memory impairment and synapse malfunctioning in the hippocampus, with abnormal enhancement of mGluR-LTD. Abnormal LTD might lead to excessive synapse elimination, whereas physiological LTD is crucial in hippocampal-dependent memory. The activation of 5-HT7R by 5-HT, or 8-OH-DPAT, or LP-211 in hippocampal slices from the FXS mouse model was able to correct excessive mGluR-LTD, bringing it back to its physiological level and thereby restoring synaptic plasticity (Costa et al., 2012). Hippocampal LTP and LTD are the most studied paradigms of synaptic plasticity that cause enduring strengthening and weakening of synapses, paralleled by increase and decrease of dendritic spine volume (Bosch and Hayashi, 2012). These data indicate that brain plasticity is accompanied by modification of neuronal connectivity and formation of new neuronal circuits. The molecular and cellular mechanisms of this modulation are still only partially known, but 5-HT7R seems a good candidate to be involved in the molecular cascade.

## The serotonin receptor 7 modulates connectivity in adolescent and mature brain

In addition to the involvement of 5-HT7R in neuronal cytoarchitecture and network construction during embryonic and early postnatal life, 5-HTR7 seems to play a role in the modulation of structural plasticity in adolescent and mature brain circuits. Indeed it is now widely accepted that mature mammalian brain undergoes dramatic structural reorganization with time and experience (Holtmaat and Svoboda, 2009; Sala and Segal, 2014). Intriguingly, it has been demonstrated that brain wiring may be modulated by chronic pharmacological intervention, as indicated by the comprehensive phenotype correction, including dendritic spine density, in adult mice models of FXS treated in young adulthood with a selective mGlu5 inhibitor (Michalon et al., 2012).

In adolescent rodents, it has been hypothesized that 5-HT7R may subserve the persistent structural rearrangements of the brain reward pathways occurring during postnatal development, following chronic methylphenidate exposure (Adriani et al., 2006; Leo et al., 2009). Accordingly, stimulation of 5-HT7R in adolescent rats by intraperitoneal administration of LP-211 (0.25 mg/kg/day for 5 days), induces plastic rearrangements within forebrain networks, accounting for long-lasting behavioral changes in the adulthood (Canese et al., 2014). Similar results were obtained in a rat model for Attention Deficit Hyperactivity Disorder (ADHD) in which prepuberal stimulation of 5-HT7R by intraperitoneal administration of LP-211 (up to 0.5 mg/kg/day for 14 days), has long-term effects on adult behavior, improving spatial attention and resulting in modified expression of pre- and post-synaptic markers (Ruocco et al., 2014a), while the same treatment, during adolescence, modulates the emotional responses (Ruocco et al., 2014b). In addition, stimulation of 5-HT7R exerted consistent effects into exploratory motivation, anxiety-related profiles and spontaneous circadian rhythm in adult rodents (Adriani et al., 2012).

Most of these experiments have been performed using LP-211, a novel highly potent and selective 5-HTR7 agonist that, in being brain-penetrant, is particularly useful for *in vivo* studies (Hedlund et al., 2010). Thus, adult mice treated *in vivo* with intraperitoneal injection of LP-211 (0.25 mg/kg/day for 3 days) showed a significant increase in the total number and density of dendritic spines in neurons of the dorso-lateral striatum (Speranza et al., in preparation for this issue). In view of the fact that dendritic spines actively participate in the formation of synapses, these data strongly support the notion that this receptor may be involved in shaping the neuronal wiring of the mature CNS.

Along this line, LP-211 stimulation of 5-HT7R by intraperitoneal administration of LP-211 (0.25 mg/kg/day for 7 days) in an adult mouse model of Rett Syndrome (the MeCP2-308 strain) was able to rescue the behavioral deficits and to reverse the abnormal activation of the key molecules regulating actin cytoskeleton dynamics, which in turn modulate neuronal morphology (De Filippis et al., 2014). In addition, inhibition of 5-HT7R with the selective antagonist SB-269970 was able to ameliorate psychomotor and cognitive deficits in animal model of schizophrenia (PACAP-deficient mice), supporting the notion that 5-HT7R is linked to the already mentioned psychiatric disorders such as schizophrenia and depression (Tajiri et al., 2012). This view has been further supported by independent experiments using lurasidone, a novel atypical antipsychotic drug with a powerful antagonist activity for 5-HT7R. Lurasidone ameliorates learning and memory deficits in animal model of schizophrenia and induces an antidepressant-like response in animal models for depression and anxiety. Interestingly, these pharmacological actions of lurasidone are mediated, at least partially, by 5-HT7R (Ishibashi et al., 2010; Cates et al., 2013; Horisawa et al., 2013), corroborating previous data that demonstrate the involvement of 5-HT7R in depression (Hedlund et al., 2005; Mnie-Filali et al., 2007).

The 5-HT7R expression in brain regions involved in learning and memory parallels with its functions. The 5-HT7R knock-out mice exhibits specific impairments in contextual learning (Roberts et al., 2004). Several other studies highlight the implication of 5-HT7R in memory and attention-related processes (Gasbarri et al., 2008; Freret et al., 2014; Meneses, 2014), underscoring its role in the modulation of the neuronal network associated with cognitive functions. Therefore, the study of this receptor and its associated intracellular pathways may provide invaluable information for the treatment of learning and memory disorders. From a general point of view, the involvement of 5-HT7R in such numerous neurological disorders associated with abnormal CNS connectivity, recognizes this receptor as a promising target for the development of innovative therapeutical strategies.

## Conclusion

Taken together the results highlighted here indicate that 5-HT7R is an important player involved in the establishment of neuronal cytoarchitecture during development of CNS, and strongly suggest its modulatory action in remodeling neuronal morphology and circuitry in the mature brain. Future studies using high resolution *in vivo* imaging, coupled with the elucidation of molecular mechanisms responsible for morphological modifications will further our knowledge on 5-HT7R role in brain plasticity.

## Conflict of interest statement

The authors declare that the research was conducted in the absence of any commercial or financial relationships that could be construed as a potential conflict of interest.
